# Simultaneous mapping of the ultrafast time and fluence dependence of the laser-induced insulator-to-metal transition in magnetite

**DOI:** 10.1063/4.0000288

**Published:** 2025-03-20

**Authors:** J. O. Schunck, P. S. Miedema, R. Y. Engel, S. Dziarzhytski, G. Brenner, N. Ekanayake, C.-F. Chang, P. Bougiatioti, F. Döring, B. Rösner, C. David, C. Schüßler-Langeheine, M. Beye

**Affiliations:** 1 Deutsches Elektronen-Synchrotron DESY, Notkestr. 85, 22607 Hamburg, Germany; 2 Physics Department, Universität Hamburg, Luruper Chaussee 149, 22761 Hamburg, Germany; 3 Max Planck Institute for Chemical Physics of Solids, Nöthnitzer Straße 40, 01187 Dresden, Germany; 4 Paul Scherrer Institut (PSI), Forschungsstraße 111, 5232 Villigen, Switzerland; 5 Helmholtz-Zentrum Berlin für Materialien und Energie, Albert-Einstein-Straße 15, 12489 Berlin, Germany; 6 Department of Physics, AlbaNova University Center, Stockholm University, SE-10691 Stockholm, Sweden

## Abstract

Pump-probe methods are a ubiquitous tool in the field of ultrafast dynamic measurements. In recent years, x-ray free-electron laser experiments have gained importance due to their ability to probe with high chemical selectivity and at atomic length scales. Measurements are typically repeated many thousands of times to collect sufficient statistics and vary parameters like delay or fluence, necessitating that initial conditions are restored each time. An alternative is presented by experiments which measure the relevant parameters in a single shot. Here, we present a time-to-space mapping imaging scheme that enables us to record a range of delays and laser fluences in any single shot of the x-ray probe. We demonstrate the use of this scheme by mapping the ultrafast dynamics of the optically induced insulator-to-metal Verwey transition in a magnetite thin film, probed by soft x-ray resonant diffraction. By extrapolating our results toward the conditions found at x-ray free-electron lasers with higher photon energy, we demonstrate that the presented data could be recorded in a single shot.

## I. INTRODUCTION

The intriguing properties of complex, functional, and quantum materials, like high-temperature superconductivity,^1,2^ colossal magnetoresistance,^3,4^ strange metallic behavior,^5,6^ and insulator-metal phase transitions^7^ have attracted researchers' interest on their quest to engineer and harvest these phenomena for several decades. Excitation with ultrashort laser pulses is a particularly powerful tool to manipulate equilibrium phases or create transient states which do not occur under equilibrium conditions.^8–11^ Usually, time-resolved studies are performed using two ultrashort pulses: the first pulse (the pump) starts a dynamic process and the subsequent pulse (the probe) is used to observe the change induced in the sample. Due to typically limited signal levels, this pump-probe process is repeated thousands or millions of times. This allows one to collect sufficient statistics while also varying the temporal separation between the two pulses (the delay) and/or the fluence of the pump pulse.

X-ray free-electron lasers (FELs) offer the opportunity to use x-ray pulses for element-selective and atomic-scale probing with high signal levels and femtosecond temporal resolutions. Many FELs today generate pulses using the process of self-amplified spontaneous emission (SASE),^12–14^ which results in fluctuating pulse properties, like intensity, pulse duration, and spectral content. This characteristic motivated the development of techniques for recording a complete set of information for every single shot. In the post-analysis, the data can then be sorted and binned appropriately. Here, simultaneous detection of a range of parameters offers the opportunity to assign recorded data to its respective state, thereby facilitating a study of such systems in the first place. In repeated pump-probe experiments, in general, one needs to take care that each pair of pulses yields the same information. This is compromised, e.g., by fluctuating pump and probe beams or jitter between the two. Furthermore, the sample needs to return to the ground state between the pulse pairs and the pulse energies should not cause permanent damage to the sample. Sometimes though, it is interesting to actually probe nonreversible excitations. In this case or if the sample is destroyed, a new sample or spot on the sample needs to be used for every measurement, posing requirements on the available amount or size of sample. Many of these challenges can be alleviated using approaches that can perform a complete experiment, i.e., measure the required delay (and potentially fluence) dependence, in a single shot.

One possible solution is an experimental setup, which is able to map a range of one or more parameters simultaneously. In the context of x-ray spectroscopy at synchrotrons, this has, for example, been done by mapping a range of incident and emitted photon energies onto an area detector.^15–17^ At FELs, one example of the mapping of pump-probe delay range is the x-ray streaking method,^18–20^ used at x-ray free-electron lasers to probe a delay range of approximately 1.5 ps in a single measurement. Also different approaches using non-colinear beams^21–24^ have been realized.

**FIG. 1.**
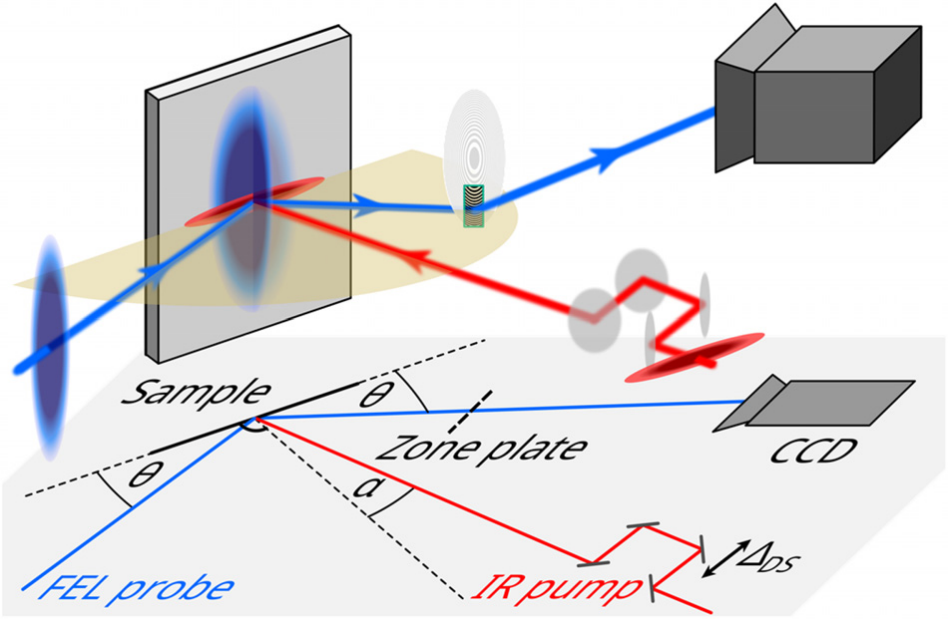
Time-to-space mapping setup. Soft x-ray pulses from FLASH resonant to the Fe *L*_3_ edge (blue) probe the resonant scattering of Fe_3_O_4_ upon pumping with 800 nm pulses (red). The horizontal scattering plane is shown in yellow. The incidence and scattering angle *θ* is 32.75° with respect to the sample surface and the pump laser is incident onto the sample under an angle *α* of 16° with respect to the sample surface normal. Due to the relative angle between FEL and pump, a delay range of several picoseconds is probed simultaneously. An additional delay $\Delta_{DS}$ can be set by a mechanical delay stage. An off-axis Fresnel zone plate images the resonant scattering of the unfocused FEL beam from the sample onto a two-dimensional CCD detector.

Here, we present the results of setup that enables the simultaneous recording of a delay range of seven picoseconds and a relative fluence range of a factor of more than five. We use an x-ray optical Fresnel zone plate to image the iron *L*_3_-edge resonant soft x-ray diffraction (RSXD) signal from a magnetite sample. A non-collinear pump-probe geometry with an angle of 73.25° between the pump and probe beams allows the pump-probe delay to be mapped onto a spatial axis on the detector (see Fig. 1). The orthogonal axis images the signal across the laser beam spot onto the detector, essentially mapping the pump laser fluence distribution. The concept of mapping a range of fluences by using zone plates have previously been reported.^25,26^ With our setup, a large parameter range can be acquired in a static geometry without scanning. Given a sufficiently high signal, such a setup even allows capturing a complete dataset in a single shot.

As a sample, we chose a thin magnetite (Fe_3_O_4_) film. Magnetite continues to serve as a model system for how charge, orbital, and lattice degrees of freedom shape an insulator–metal transition, here called Verwey transition:^27–40^ Below 124 K, the resistivity increases by two orders of magnitude due to a structural transition from a high-temperature cubic inverse-spinel structure to a distorted monoclinic structure,^41^ characterized by charge and orbital order (COO).^35,36,38–40^ The excitation of the magnetite low-temperature phase with ultrashort infrared (IR) laser pulses destroys the COO superstructure and drives the system into the high-temperature structure within several picoseconds.^42–47^ Previous time-resolved IR pump, RSXD probe studies^42,43^ on magnetite bulk single crystals observed a two-step process on femto- and picosecond time scales, characterized by an initial sub-picosecond reduction of the scattering intensity caused by direct excitation of charge transfer between charge ordered sites.^33,44^

## II. METHODS

As outlined in the Introduction, a non-colinear geometry of FEL and pump laser is realized in our time-to-space mapping setup, schematically sketched in Fig. 1: Due to the angle between the two beams, the relative pulse front arrival time of both pulses varied along the horizontal dimension, thereby mapping a time (i.e., relative delay) axis to the horizontal space coordinate of the sample. With an off-axis Fresnel zone plate (FZP), the resonant diffraction signal from the sample, including this delay axis, was spatially resolved by imaging onto a two-dimensional charge coupled device (CCD). The delay range which can be covered in a single shot was determined by the geometry of the experiment (incident angles of FEL and laser beams), as well as the magnification of the FZP and the properties of the detector. In the present setup, the delay axis was mapped on the CCD as 23.6 fs/pixel. In principle, this would allow us to record a delay range of up to 50 ps, but in our experiment, the simultaneously recorded delay range was limited by the horizontal sample size to approximately 7 ps. Like in a conventional pump-probe scheme, the recorded delay range can be further extended by scanning the pump laser delay stage ( $\Delta_{DS}$ in Fig. 1).

Both the FEL and the probe laser illuminate an extended area on the order of one square millimeter on the sample, necessitating a homogeneous sample with flat high-quality surface is required. Here, a high-quality magnetite thin film was used. In addition to being flat, thin films epitaxially grown on spinel Co_2_TiO_4_ substrates that closely match the crystal structure and lattice constant of magnetite have been shown to have excellent properties:^48^ The resistance hysteresis width of the Verwey transition matches that of the bulk, the correlation length of the COO is comparable to bulk single crystals, and the Verwey transition temperature even surpasses that of the bulk by 3 K.

In order to keep the laser profile rather homogeneous along the probed horizontal direction, which is used as delay axis, we used a cylindrical lens to focus the optical laser to a horizontal line on the sample. In the vertical direction, the laser is focused much stronger, well below the size of the probing FEL beam, such that the full vertical spatial laser profile was probed simultaneously. In the data analysis, we could, thus, separate regions in the center of the vertical laser spot profile, which were pumped with a high local fluence, from more weakly pumped regions further away.

**FIG. 2.**
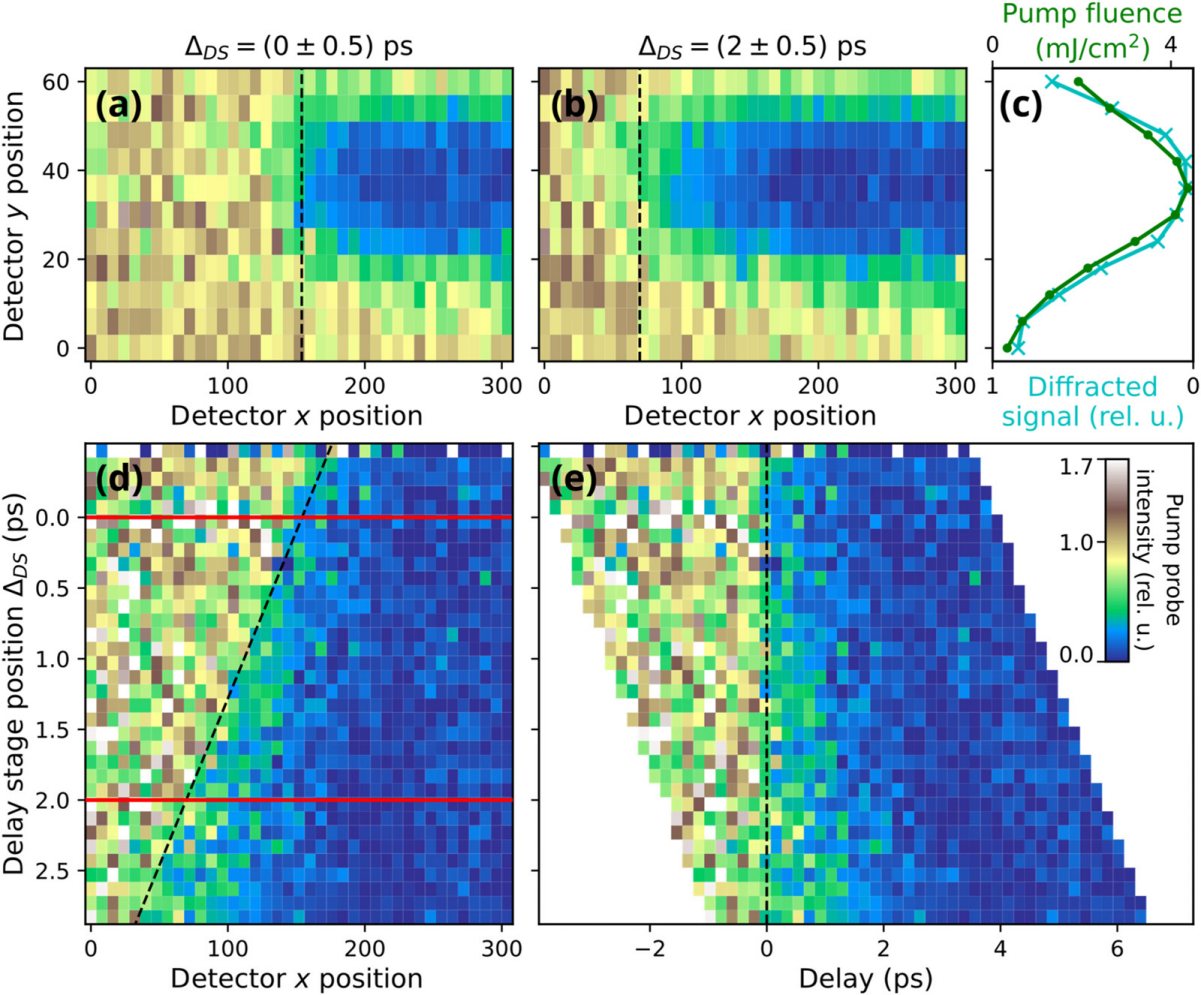
Pump-probe data treatment. The pump-probe delay varies along the horizontal x axis because of the different incidence angles of FEL and pump laser. Panels (a) and (b): Normalized images of the pump effect on the sample obtained by dividing pumped by unpumped events for delay stage position ranges of $\Delta_{DS}=$ (0 ± 0.5) and $\Delta_{DS}=$ (2 ± 0.5) ps, respectively. One positional unit on the detector corresponds to 13.5 *μ*m. Panel (c) shows the laser beam profile (green, upper axis) and the corresponding profile of the diffracted signal (cyan, lower axis), obtained by averaging only the pumped signal in panel (b) along the horizontal axis. Panel (d): By scanning the pump laser delay stage position $\Delta_{DS}$ (see also Fig. 1) and plotting the average of detector *y* positions pumped with the highest fluences against the delay stage position [data shown along the red lines are extracted from panels (a) and (b)] allows one to observe how temporal overlap moves along *x* (black dashed line). The slope of the black dashed line can be used to translate every row onto a common delay axis. The x axis and y axis have been binned by a factor of 8 and 6, respectively. Panel (e) then shows the dataset on the common horizontal delay axis, while the vertical axis still shows the mechanical delay stage position. Pump-probe traces, as shown in Fig. 3, are finally derived by averaging the data in panel (e) along the vertical dimension.

Our imaging setup recorded two spatial dimensions simultaneously. We used our special experimental configuration to map the spatial coordinates onto one temporal and one local fluence axis. As this scheme introduces a correlation of signals recorded with different delays and fluences with sample position, it works best with samples that are spatially homogeneous over the probed area. If dynamics of a non-homogeneous samples are of interest, our setup has the advantage of parallelizing the data acquisition in comparison to a conventional pump-probe scheme. In such a conventional scheme, inhomogeneities would be studied by focusing the FEL to a spot as small as possible (with damage-related limitations to the usable total number of photons). Subsequently, a full dataset would require a four-dimensional scan of pump-probe delay, pump laser fluence, and the two lateral dimensions of the sample. In this case, our setup reduces the required scanned dimensions from four to the two spatial dimensions. The ability to study inhomogeneities is closely tied to the spatial resolution of our setup, which we estimate to be around 3–4 *μ*m for the present geometry^16^ (see also Sec. III). In the present experiment, we scanned the relative pump-probe delay $\Delta_{DS}$ (see Fig. 1) back and forth by 3 ps using a movable delay stage, thereby moving the entire 7 ps delay window horizontally over the sample surface. This scan also served as a cross-calibration of the delay axis (see below and Fig. 2).

## III. EXPERIMENTAL SETUP

Measurements with the time-to-space mapping setup were performed at beamline BL2^49–51^ of the free-electron laser (FEL) FLASH at DESY in Hamburg, Germany, using the ultrahigh-vacuum diffractometer MUSIX.^52^ The schematic experimental setup is shown in Fig. 1. To record resonant diffraction from a magnetite sample at the iron *L*_3_-edge at FLASH, third harmonic radiation with a central photon energy of 706 eV was used. The full width at half-maximum (FWHM) bandwidth of the FEL pulses was approximately 3.6 eV. The pulse energy of the FEL fundamental was on average 20 *μ*J and the third harmonic emission is usually two to three orders of magnitude weaker.^53–55^

The unfocused FEL beam had a diameter of approximately 4.2 mm at the sample position. The pump laser^56^ wavelength was centered around 800 nm, the pulse duration was 57 fs FWHM, and the pulse energy was 16.8 *μ*J. The combined temporal resolution of the experiment was ∼120 fs (see the supplementary material).^61^ The laser spot measured approximately 2.6 mm (horizontal, FWHM) by 0.2 mm (vertical, FWHM) and was focused such that a flat-top region formed in the horizontal direction (see the supplementary material, Fig. S1).^61^ The sample was horizontally smaller than the laser footprint, extending only in the flat-top region of the laser focus. The pulse repetition rate of the FEL was 10 Hz, while that of the pump laser was 5 Hz so every other FEL shot probed the unpumped sample, serving as a reference for normalization of the signal. We verified that the sample was fully recovered in between shots. Due to the small third harmonic fraction of the FEL beam, the scattering cross section of the sample and the large FEL footprint, the count rate for the dataset was on average 0.54 counts/shot. In total, the data shown here comprises 12 h of acquisition time and approximately 230 000 counts.

The pump laser beam was incident under an angle of 16° with respect to the sample surface normal (see Fig. 1). The sample was a 40 nm thin magnetite (Fe_3_O_4_) film, grown on Cr_2_TiO_4_ (001),^48^ measuring approximately 1.9 mm (horizontal) by 8 mm (vertical). The incidence angle *θ* of the FEL was 32.75° with respect to the sample surface to fulfill the Bragg condition for the (00½) superstructure peak. The sample was cooled to a base temperature of 100 K for the entire duration of the measurements. An off-axis Fresnel zone plate (FZP) imaged the diffraction signal onto a two-dimensional charge-coupled device (CCD), thereby magnifying the diffraction signal from the sample by a factor of 4.1. Further details on the experimental setup can be found in the supplementary material.^61^

## IV. RESULTS

As described in Sec. II, we obtained data as a function of the horizontal sample position, *x*, the vertical sample position, *y*, and the delay stage position, $\Delta_{DS}$. How we obtain the calibration of the delay axis on the detector coordinates from this multidimensional dataset, as well as data treatment steps used to obtain fluence-dependent delay traces, are visualized in Fig. 2. Small regions of the sample imaged at delay stage positions $\Delta_{DS}$ of 0 ± 0.5 and 2 ± 0.5 ps are shown in panels (a) and (b), respectively. In both panels, the depicted pump signal has been normalized to the unpumped signal. The part of the sample where the COO was pumped away by the laser is visible as the blue area. The horizontal position where both pulses cross the surface at the same time (time overlap, *t*_0_) can be seen to be the *x* position where the signal transitions from high to low scattering intensity (black dashed lines). We chose the origin of the $\Delta_{DS}$ scale such that *t*_0_ is in the horizontal center of the detector window (*x* = 150 px) [see Fig. 2(a)].

The vertical profile of the signal which is averaged in the pumped area of panel (b) along the x axis is shown as turquoise data in panel (c) of Fig. 2. This profile maps the local strength of the pump-probe effect and follows the local fluence profile of the laser, which we show overlaid to the pump laser profile as extracted from an independent knife edge scan (green).

We now focus on calibrating the delay scale. For this, we use the region with the largest pump effect in the vertical center of the laser spot profile. We average the *x*-traces with pump fluences higher than 4 mJ/cm^2^ and plot these for the different delay stage positions as a map [Fig. 2(d)]. *t*_0_ extends along a diagonal (dashed line) reflecting the linear influences of both *x* and $\Delta_{DS}$ on the delay. The slope of this diagonal line yields the delay mapping factor on our detector (here 23.6 fs/px). This value depends on the relative incidence angle between FEL and pump laser, the sample and observation angles, the magnification of the imaging zone plate, and the pixel size of the detector. By choosing these parameters, the setup-limited temporal resolution can be tuned to match the experimental requirements.

With this conversion, we can align each row to a common delay axis [see Fig. 2(e)]. A common delay trace using all available data are then obtained by averaging the data in Fig. 2(e) along the *y* axis. We note that there is a hint toward a small sample position dependence of the observed pump effect; it appears that the sample region that is mapped onto *x* ≈ 90 exhibits a somewhat slower pump dynamics, which can be seen as a comparatively higher intensity around a delay value of 1 ps for delay stage positions of approximately 2.5 ps. However, the present dataset does not have sufficient quality to study this rather weak effect in more detail.

**FIG. 3.**
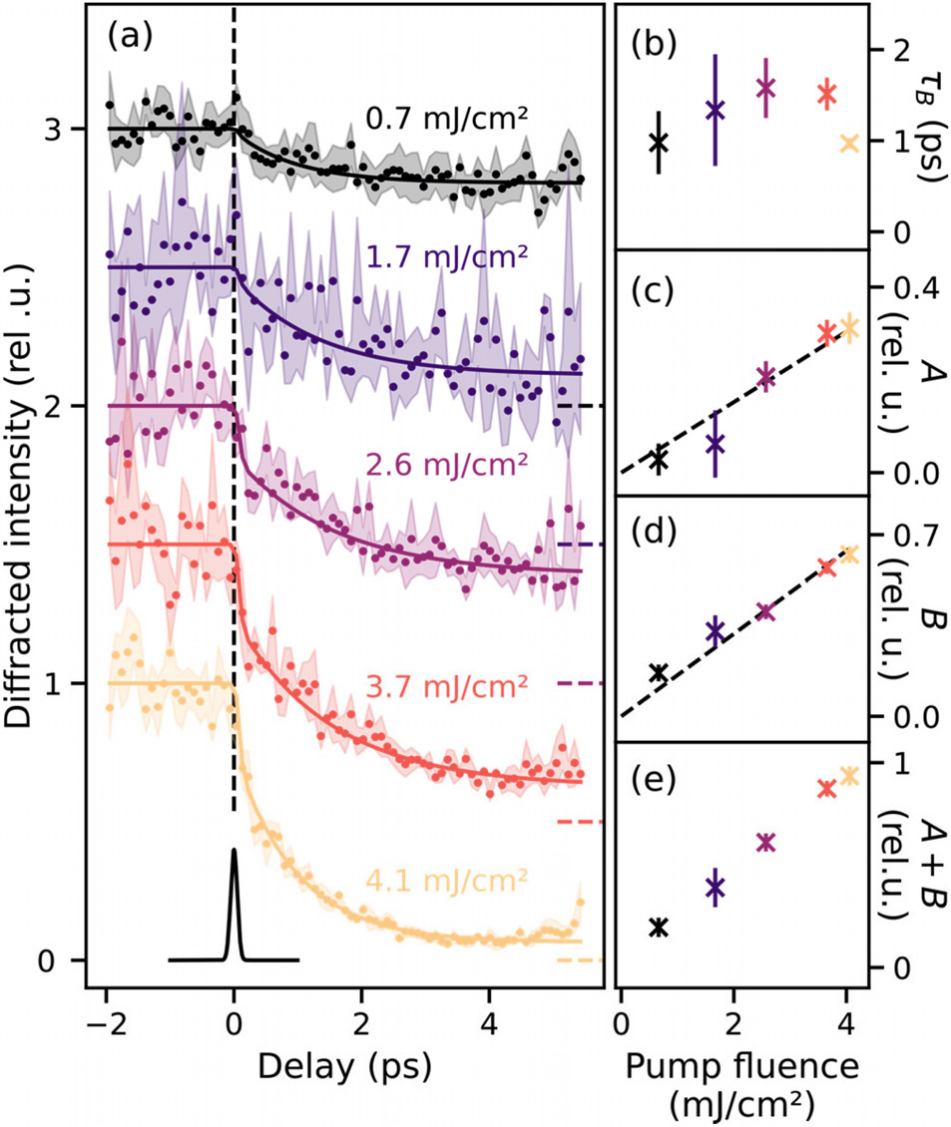
Ultrafast fluence-dependent response of a 40 nm thin magnetite film to 800 nm laser excitation. Panel (a): The intensity of the (00½) superstructure peak decreases on a few-picosecond timescale upon irradiation with the pump laser for fluences between 0.7 and 4.1 mJ/cm^2^. The data were normalized to 1 for negative delays and fit curves were offset by 0.5 relative units (rel. u.) for clarity, and the dashed lines to the right show 0 intensity for the data of the respective fluence. The shaded area is the one-sigma measurement uncertainty of the data, calculated as the standard deviation for each delay value upon averaging several traces of similar fluence and binning on the delay axis. Full lines show fits with a double exponential decay convolved with a Gaussian profile with a width corresponding to the experimental resolution, shown below in black at a delay of 0 ps. The fast time constant $\tau_{A}$ is set to be much faster than the temporal resolution. The fluence dependence of the slower time constant $\tau_{B}$ is shown in panel (b). Panels (c) and (d): Fluence dependence of the amplitudes A (fast process) and B (slow process) alongside linear trend lines with slopes 0.08 and 0.16 rel. u./(mJ/cm^2^), respectively. Panel (e): The total decay amplitude (i.e., sum of *A* and *B*). See the supplementary material for details on the fitting procedure.^61^

Usually, the pump-fluence dependence of the ultrafast response of the sample to IR excitation is determined by repeating the pump-probe experiment with different pump laser attenuation settings. In our setup, we can do this by sorting different rows of the detector image according to the pump fluence at that position [see Fig. 2(c)]. We chose to sort the data into five fluence bins (see the supplementary material; Fig. S2).^61^ Thus, we obtain five delay traces spanning a fluence range between 0.7 and 4.1 mJ/cm^2^ without actually changing the incident laser pulse energy. The resulting fluence-dependent IR pump and RSXD probe traces are depicted in Fig. 3(a), showing a double exponential decay characterized by a fast and a slow component.

The resulting traces were fitted using a double exponential decay function (solid lines) convolved with a Gaussian of a width corresponding to the temporal resolution of the experiment (120 fs FWHM) as a fit model. The shaded regions in Fig. 3(a) show the experimental one-sigma standard deviation calculated for each data point during the averaging of multiple traces and binning on the x axis. During the weighted least squares fitting, the inverse experimental error is used as weights for the respective data points. We find that following the IR excitation, a fast and a slow decay process, respectively, named *A* and *B*, occur, matching what has previously been observed for single crystals.^42,43,57^ Within our experimental uncertainty, there are no indications of a recovery in the measured delay range.

In panels (b)–(e) of Fig. 3, the fluence dependence of the fit results for the slow decay time constant $\tau_{B}$ (b), the amplitudes *A* (c) and *B* (d) and the total long-time decrease *A* + *B* (e) are shown. Error bars represent the one-sigma standard deviations. The fast decay is limited by the temporal resolution of the experiment to 120 fs. In the fitting procedure, this is realized by the convolution with the resolution-limited Gaussian function; therefore, the value of $\tau_{A}$ was fixed to a value of 10 fs, much faster than the temporal resolution. Both amplitudes, *A* and *B*, show a linear increase with fluence [black dashed lines in Figs. 3(b) and 3(c)]. The slow decay time constant $\tau_{B}$ ranges from 1 to 2 ps, generally matching previously reported values.^43^ See the supplementary material for more details on the fitting procedure.^61^

## V. DISCUSSION

In the data shown before, we analyze the temporal evolution of the intensity of the (00½) superstructure peak, which is a direct measure for the degree of charge and orbital order in magnetite. In the low-temperature ordered phase, this reflection appears in resonant soft x-ray diffraction (RSXD), e.g., at the iron *L_2,3_*-edges and disappears when the sample transforms into the high-temperature phase, indicating the disappearing charge and orbital order.^29,30,39,40,58^ The IR pump laser can drive this phase transition and, thus, the (00½) reflection vanishes as a function of pump-probe delay. The IR pump and RSXD probe results obtained on magnetite thin films qualitatively match those previously reported for single crystals:^42,43,57^ we find a biexponential decay with a fast time constant $\tau_{A}$ limited by the 120 fs temporal resolution and a slow time constant on the picosecond scale. The linear fluence dependence of amplitude A, previously reported for single crystals,^43^ is confirmed by our measurements. Quantitative differences beyond these generally agreeing results are attributed to differing experimental conditions: The fact that we do not observe two distinct fluence regimes is because the lowest fluence shown in Fig. 3(a) is already sufficient to drive the sample from its initial temperature of 100 K through the Verwey transition at 124 K. With a higher signal level, our results could have been binned finer to allow drawing conclusions also on the lower fluence regime. Furthermore, the fluence dependence of the fast decay amplitude A has been reported to scale as 0.3 rel.u./(mJ/cm^2^) for single crystals,^43^ while we find 0.08 rel.u./(mJ/cm^2^) for the thin-films studied here.

This dependency likely arises because a comparison of fluences in different experiments is challenging. Small deviations coming from differing calibrations in spot sizes and laser pulse energies, different estimates of sample reflectivity (that also relate to surface quality) as well as using different incidence angles and polarizations may add up to sizeable differences. For a quantitative comparison, one would ideally study both sample types in the same setup. As intrinsic sample properties are concerned, differences between our magnetite thin film and a bulk magnetite crystal are expected to be small: A study directly comparing static RSXD and x-ray absorption data of thin films and bulk single crystals only finds minor spectral differences.^58^ The limited film thickness is likely to not explain this behavior, since the x-ray probing depth of around 10–40 nm (Refs. 42 and 43) is shorter than the film thickness. Different thermal conductivities of the film substrate as compared to the bulk sample are expected to influence the evolution only on longer timescales than studied here.

Having discussed the observed dynamics in magnetite, we now turn to our time-to-space mapping method. In its current implementation, signal levels of the setup were limited to approximately 0.5 counts/shot on average. Within an acquisition time of 12 h, we collected about 230 000 shots from the FEL, comprising the data shown in Fig. 3. Mainly two aspects contribute to this rather low count rate: The used pulses from FLASH had 20 *μ*J pulse energy in the fundamental with an expected contribution at the Fe *L*-edge of about 20 nJ. Additionally, focusing the FEL beam to a round spot with about 4.2 mm diameter results in a loss of photons of more than one order of magnitude, if compared to the actually relevant photons probing the pumped area of the sample which measured $2\times 0.2\;mm^{2}$. Improving these two aspects could, thus, gain a more than four orders of magnitude in signal. If additionally higher-energy FELs, like the European XFEL, PALFEL, SwissFEL, or LCLS, which can typically produce mJ level pulse energies at these photon energies,^59^ were used, a total gain of about six orders of magnitude could be realized in an optimized experiment. With these improvements, the signal level of a single shot has the potential to substantially surpass that of the full dataset presented here meaning that, our setup can be used in the future to record full datasets on laser-induced dynamics of sensitive samples in a single shot with complete information on time- and fluence dependences.

We note though that the conditions described above result in probe fluences of about 500 mJ/cm^2^, which is certainly beyond the damage threshold of the majority of solids. Consequently, single-shot acquisition becomes a necessity in a measure-before-destroy approach. In order to reduce the probe fluence to a level of <1 mJ/cm^2^, which should be tolerable and non-disturbing to most samples,^60^ the FEL pulse could be attenuated by about one order of magnitude while still yielding a dataset of comparable quality to that shown here. The remaining about two orders of magnitude can be recovered by increasing the probe spot size, for example, by using an FEL spot size of $6\times 6\;mm^{2}$. While solid samples and sample holders of this size are not uncommon, this size requirement may pose a challenge if e.g., homogeneous magnetic fields are required to prepare certain sample states. An x-ray focus of this size is routinely achievable with bendable KB-optics which are relatively common at modern FEL facilities. Consequently, the spot size of the optical pump laser would need to be increased accordingly, necessitating an increase in pulse energy to adequately excite the sample.

If these requirements can be fulfilled, the current delay and fluence ranges which are probed simultaneously can be increased. In its current implementation, the setup probes a delay range of 7 ps and a fluence range of more than a factor of five in a single acquisition. The delay range depends on the size of the horizontal pump laser-FEL overlap and the angle between the two. An overlap range of 6 mm, as discussed above, would increase the observed delay range to 21 ps. The current distribution of fluence bins (see Fig. 3 and the supplementary material^61^) is currently restricted by the limited signal level. Improvements in signal would enable a finer binning of the laser fluence profile and also allow to increase the probed fluence range to lower fluences.

## VI. CONCLUSION AND OUTLOOK

In this study, we present results of a time-to-space mapping setup for time-resolved pump-probe experiments. With this setup, the ultrafast phase transition dynamics of high-quality magnetite (Fe_3_O_4_) thin films was investigated, induced by 800 nm laser pulses using resonant soft x-ray diffraction at the iron *L*_3_-edge as probe. The induced dynamics generally match those previously observed in single crystals.

This setup is capable of recording a range of delays (dependent on the dimensions of the sample and the laser and FEL beams) and fluences in a static setup, without the need for any scans. This is achieved within a range of a few millimeters on the sample where the pump laser and FEL overlap, both incident under a relative angle of 73.25°. Due to this angle, the relative delay of both pulses is imprinted into the horizontal dimension on the sample and imaged onto the detector with a Fresnel zone plate. In the vertical direction, imaging the laser spot profile allows for the simultaneous probing of regions of the sample that have been pumped by higher (in the center of the spot) or lower (at the edges of the spot) fluences.

In addition to its use for resonant scattering experiments, the method presented in this work for mapping of delay and fluence ranges can be transferred to other x-ray spectroscopy schemes, like non-resonant scattering, x-ray absorption measured in transmission or reflectivity, and potentially x-ray magnetic circular dichroism. The resonant diffraction efficiency of magnetite at the iron *L*_3_-edge is about $10^{-4},$^57^ which is small in comparison to e.g., absorption measured in transmission. Since the necessary sample and spot sizes as well as pulse energies critically depend on the signal level, other detection modalities will change the feasibility of this approach (see Sec. V).

Acquisition schemes similar to our method could also be realized in laboratory-based experiments using high-harmonic generation (HHG). While such a source would not provide enough photons for single-shot acquisitions, the stability together with our non-scanning approach may yield excellent data quality after averaging.

## Data Availability

The data that support the findings of this study are available from the corresponding author upon reasonable request.

## References

[1] J. G. Bednorz and K. A. Müller, “Possible high T_c_ superconductivity in the Ba-La-Cu-O system,” Z. Phys. B 64, 189–193 (1986). 10.1007/BF01303701

[2] B. Keimer, S. A. Kivelson, M. R. Norman, S. Uchida, and J. Zaanen, “From quantum matter to high-temperature superconductivity in copper oxides,” Nature 518, 179–186 (2015). 10.1038/nature14165 25673411

[3] E. Dagotto, T. Hotta, and A. Moreo, “Colossal magnetoresistant materials: The key role of phase separation,” Phys. Rep. 344, 1–153 (2001). 10.1016/S0370-1573(00)00121-6

[4] E. Dagotto, “Complexity in strongly correlated electronic systems,” Science 309, 257–262 (2005). 10.1126/science.1107559 16002608

[5] H. Takagi, B. Batlogg, H. L. Kao, J. Kwo, R. J. Cava, J. J. Krajewski, and W. F. Peck, “Systematic evolution of temperature-dependent resistivity in La_2−x_Sr_x_CuO_4_,” Phys. Rev. Lett. 69, 2975–2978 (1992). 10.1103/PhysRevLett.69.2975 10046689

[6] P. W. Phillips, N. E. Hussey, and P. Abbamonte, “Stranger than metals,” Science 377, eabh4273 (2022). 10.1126/science.abh4273 35857547

[7] M. Imada, A. Fujimori, and Y. Tokura, “Metal-insulator transitions,” Rev. Mod. Phys. 70, 1039–1263 (1998). 10.1103/RevModPhys.70.1039

[8] J. Orenstein, “Ultrafast spectroscopy of quantum materials,” Phys. Today 65(9), 44–50 (2012). 10.1063/PT.3.1717

[9] M. Mitrano and Y. Wang, “Probing light-driven quantum materials with ultrafast resonant inelastic X-ray scattering,” Commun. Phys. 3, 184 (2020). 10.1038/s42005-020-00447-6

[10] A. de la Torre, D. M. Kennes, M. Claassen, S. Gerber, J. W. McIver, and M. A. Sentef, “Colloquium: Nonthermal pathways to ultrafast control in quantum materials,” Rev. Mod. Phys. 93, 041002 (2021). 10.1103/RevModPhys.93.041002

[11] A. Zong, B. R. Nebgen, S.-C. Lin, J. A. Spies, and M. Zuerch, “Emerging ultrafast techniques for studying quantum materials,” Nat. Rev. Mater. 8, 224–240 (2023). 10.1038/s41578-022-00530-0

[12] A. M. Kondratenko and E. L. Saldin, “Generation of coherent radiation by a relativistic electron beam in an ondulator,” Part. Accel. 10, 207–216 (1980).

[13] S. V. Milton, E. Gluskin, N. D. Arnold, C. Benson, W. Berg, S. G. Biedron, M. Borland, Y.-C. Chae, R. J. Dejus, P. K. Den Hartog, B. Deriy, M. Erdmann, Y. I. Eidelman, M. W. Hahne, Z. Huang, K.-J. Kim, J. W. Lewellen, Y. Li, A. H. Lumpkin, O. Makarov, E. R. Moog, A. Nassiri, V. Sajaev, R. Soliday, B. J. Tieman, E. M. Trakhtenberg, G. Travish, I. B. Vasserman, N. A. Vinokurov, X. J. Wang, G. Wiemerslage, and B. X. Yang, “Exponential gain and saturation of a self-amplified spontaneous emission free-electron laser,” Science 292, 2037–2041 (2001). 10.1126/science.1059955 11358995

[14] J. Rönsch-Schulenburg, E. Hass, N. M. Lockmann, T. Plath, M. Rehders, J. Roßbach, G. Brenner, S. Dziarzhytski, T. Golz, H. Schlarb, B. Schmidt, E. Schneidmiller, S. Schreiber, B. Steffen, N. Stojanovic, S. Wunderlich, and M. Yurkov, “Operation of FLASH with short SASE-FEL radiation pulses,” in *Proceedings 36th International Free Electron Laser Conference (FEL 2014)* (JACoW, 2014), pp. 342–345.

[15] F. Marschall, Z. Yin, J. Rehanek, M. Beye, F. Döring, K. Kubiček, D. Raiser, S. T. Veedu, J. Buck, A. Rothkirch, B. Rösner, V. A. Guzenko, J. Viefhaus, C. David, and S. Techert, “Transmission zone plates as analyzers for efficient parallel 2D RIXS-mapping,” Sci. Rep. 7, 8849 (2017). 10.1038/s41598-017-09052-0 28821805 PMC5562906

[16] J. O. Schunck, F. Döring, B. Rösner, J. Buck, R. Y. Engel, P. S. Miedema, S. K. Mahatha, M. Hoesch, A. Petraru, H. Kohlstedt, C. Schüßler-Langeheine, K. Rossnagel, C. David, and M. Beye, “Soft x-ray imaging spectroscopy with micrometer resolution,” Optica 8, 156 (2021). 10.1364/OPTICA.405977

[17] V. N. Strocov, “Concept of a spectrometer for resonant inelastic X-ray scattering with parallel detection in incoming and outgoing photon energies,” J. Synchrotron Radiat. 17, 103–106 (2010). 10.1107/S0909049509051097 20029118

[18] C. David, P. Karvinen, M. Sikorski, S. Song, I. Vartiainen, C. J. Milne, A. Mozzanica, Y. Kayser, A. Diaz, I. Mohacsi, G. A. Carini, S. Herrmann, E. Färm, M. Ritala, D. M. Fritz, and A. Robert, “Following the dynamics of matter with femtosecond precision using the X-ray streaking method,” Sci. Rep. 5, 7644 (2015). 10.1038/srep07644 25561027 PMC4284506

[19] M. Buzzi, M. Makita, L. Howald, A. Kleibert, B. Vodungbo, P. Maldonado, J. Raabe, N. Jaouen, H. Redlin, K. Tiedtke, P. M. Oppeneer, C. David, F. Nolting, and J. Lüning, “Single-shot monitoring of ultrafast processes via x-ray streaking at a free electron laser,” Sci. Rep. 7, 7253 (2017). 10.1038/s41598-017-07069-z 28775262 PMC5543111

[20] E. Jal, M. Makita, B. Rösner, C. David, F. Nolting, J. Raabe, T. Savchenko, A. Kleibert, F. Capotondi, E. Pedersoli, L. Raimondi, M. Manfredda, I. Nikolov, X. Liu, A. E. D. Merhe, N. Jaouen, J. Gorchon, G. Malinowski, M. Hehn, B. Vodungbo, and J. Lüning, “Single-shot time-resolved magnetic x-ray absorption at a free-electron laser,” Phys. Rev. B 99, 144305 (2019). 10.1103/PhysRevB.99.144305

[21] W. F. Schlotter, J. Lüning, R. Rick, K. Chen, A. Scherz, S. Eisebitt, C. M. Günther, W. Eberhardt, O. Hellwig, and J. Stöhr, “Extended field of view soft x-ray Fourier transform holography: Toward imaging ultrafast evolution in a single shot,” Opt. Lett. 32, 3110 (2007). 10.1364/OL.32.003110 17975613

[22] T. Maltezopoulos, S. Cunovic, M. Wieland, M. Beye, A. Azima, H. Redlin, M. Krikunova, R. Kalms, U. Frühling, F. Budzyn, W. Wurth, A. Föhlisch, and M. Drescher, “Single-shot timing measurement of extreme-ultraviolet free-electron laser pulses,” New J. Phys. 10, 033026 (2008). 10.1088/1367-2630/10/3/033026

[23] M. Beye, O. Krupin, G. Hays, A. H. Reid, D. Rupp, S. De Jong, S. Lee, W. S. Lee, Y. D. Chuang, R. Coffee, J. P. Cryan, J. M. Glownia, A. Föhlisch, M. R. Holmes, A. R. Fry, W. E. White, C. Bostedt, A. O. Scherz, H. A. Durr, and W. F. Schlotter, “X-ray pulse preserving single-shot optical cross-correlation method for improved experimental temporal resolution,” Appl. Phys. Lett. 100, 121108 (2012). 10.1063/1.3695164

[24] H. N. Chapman, C. Li, S. Bajt, M. Butola, J. L. Dresselhaus, D. Egorov, H. Fleckenstein, N. Ivanov, A. Kiene, B. Klopprogge, V. Kremling, P. Middendorf, D. Oberthuer, M. Prasciolu, T. E. S. Scheer, J. Sprenger, J. C. Wong, O. Yefanov, M. Zakharova, and W. Zhang, “Convergent-beam attosecond x-ray crystallography,” Struct. Dyn. 12, 014301 (2025). 10.1063/4.0000275 39816474 PMC11731522

[25] M. Schneider, C. M. Günther, B. Pfau, F. Capotondi, M. Manfredda, M. Zangrando, N. Mahne, L. Raimondi, E. Pedersoli, D. Naumenko, and S. Eisebitt, “In situ single-shot diffractive fluence mapping for X-ray free-electron laser pulses,” Nat. Commun. 9, 214 (2018). 10.1038/s41467-017-02567-0 29335602 PMC5768711

[26] G. Mercurio, J. Chalupský, I.-T. Nistea, M. Schneider, V. Hájková, N. Gerasimova, R. Carley, M. Cascella, L. Le Guyader, L. Mercadier, J. Schlappa, K. Setoodehnia, M. Teichmann, A. Yaroslavtsev, T. Burian, V. Vozda, L. Vyšín, J. Wild, D. Hickin, A. Silenzi, M. Stupar, J. Torben Delitz, C. Broers, A. Reich, B. Pfau, S. Eisebitt, D. La Civita, H. Sinn, M. Vannoni, S. G. Alcock, L. Juha, and A. Scherz, “Real-time spatial characterization of micrometer-sized X-ray free-electron laser beams focused by bendable mirrors,” Opt. Express 30, 20980 (2022). 10.1364/OE.455948 36224830

[27] E. J. W. Verwey, “Electronic conduction of magnetite (Fe_3_O_4_) and its transition point at low temperatures,” Nature 144, 327 (1939). 10.1038/144327b0

[28] E. J. W. Verwey and P. W. Haayman, “Electronic conductivity and transition point of magnetite (“Fe_3_O_4_”),” Physica 8, 979–987 (1941). 10.1016/S0031-8914(41)80005-6

[29] A. Tanaka, C. F. Chang, M. Buchholz, C. Trabant, E. Schierle, J. Schlappa, D. Schmitz, H. Ott, P. Metcalf, L. H. Tjeng, and C. Schüßler-Langeheine, “Symmetry of orbital order in Fe_3_O_4_ studied by Fe L_2,3_ resonant x-ray diffraction,” Phys. Rev. Lett. 108, 227203 (2012). 10.1103/PhysRevLett.108.227203 23003646

[30] A. Tanaka, C. F. Chang, M. Buchholz, C. Trabant, E. Schierle, J. Schlappa, D. Schmitz, H. Ott, P. Metcalf, L. H. Tjeng, and C. Schüßler-Langeheine, “Analysis of charge and orbital order in Fe_3_O_4_ by Fe L_2,3_ resonant x-ray diffraction,” Phys. Rev. B 88, 195110 (2013). 10.1103/PhysRevB.88.195110 23003646

[31] M. Hoesch, P. Piekarz, A. Bosak, M. Le Tacon, M. Krisch, A. Kozłowski, A. M. Oleś, and K. Parlinski, “Anharmonicity due to electron-phonon coupling in magnetite,” Phys. Rev. Lett. 110, 207204 (2013). 10.1103/PhysRevLett.110.207204 25167445

[32] A. Bosak, D. Chernyshov, M. Hoesch, P. Piekarz, M. Le Tacon, M. Krisch, A. Kozłowski, A. M. Oleś, and K. Parlinski, “Short-range correlations in magnetite above the verwey temperature,” Phys. Rev. X 4, 011040 (2014). 10.1103/PhysRevX.4.011040

[33] S. K. Park, T. Ishikawa, and Y. Tokura, “Charge-gap formation upon the Verwey transition in Fe_3_O_4_,” Phys. Rev. B 58, 3717–3720 (1998). 10.1103/PhysRevB.58.3717

[34] J. P. Wright, J. P. Attfield, and P. G. Radaelli, “Long Range Charge Ordering in Magnetite Below the Verwey Transition,” Phys. Rev. Lett. 87, 266401 (2001). 10.1103/PhysRevLett.87.266401 11800847

[35] F. Walz, “The Verwey transition - A topical review,” J. Phys. Condens. Matter 14, 285–340 (2002). 10.1088/0953-8984/14/12/203

[36] J. P. Wright, J. P. Attfield, and P. G. Radaelli, “Charge ordered structure of magnetite Fe_3_O_4_ below the Verwey transition,” Phys. Rev. B 66, 214422 (2002). 10.1103/PhysRevB.66.214422

[37] J. García and G. Subías, “The Verwey transition—A new perspective,” J. Phys.: Condens. Matter 16, R145–R178 (2004). 10.1088/0953-8984/16/7/R01

[38] I. Leonov, A. N. Yaresko, V. N. Antonov, M. A. Korotin, and V. I. Anisimov, “Charge and orbital order in Fe_3_O_4_,” Phys. Rev. Lett. 93, 146404 (2004). 10.1103/PhysRevLett.93.146404 15524820

[39] D. J. Huang, H.-J. Lin, J. Okamoto, K. S. Chao, H.-T. Jeng, G. Y. Guo, C.-H. Hsu, C.-M. Huang, D. C. Ling, W. B. Wu, C. S. Yang, and C. T. Chen, “Charge-orbital ordering and Verwey transition in magnetite measured by resonant soft x-ray scattering,” Phys. Rev. Lett. 96, 096401 (2006). 10.1103/PhysRevLett.96.096401 16606287

[40] J. Schlappa, C. Schüßler-Langeheine, C. F. Chang, H. Ott, A. Tanaka, Z. Hu, M. W. Haverkort, E. Schierle, E. Weschke, G. Kaindl, and L. H. Tjeng, “Direct observation of t_2g_ orbital ordering in magnetite,” Phys. Rev. Lett. 100, 026406 (2008). 10.1103/PhysRevLett.100.026406 18232896

[41] M. S. Senn, J. P. Wright, and J. P. Attfield, “Charge order and three-site distortions in the Verwey structure of magnetite,” Nature 481, 173–176 (2012). 10.1038/nature10704 22190035

[42] N. Pontius, T. Kachel, C. Schüßler-Langeheine, W. F. Schlotter, M. Beye, F. Sorgenfrei, C. F. Chang, A. Föhlisch, W. Wurth, P. Metcalf, I. Leonov, A. Yaresko, N. Stojanovic, M. Berglund, N. Guerassimova, S. Düsterer, H. Redlin, and H. A. Dürr, “Time-resolved resonant soft x-ray diffraction with free-electron lasers: Femtosecond dynamics across the Verwey transition in magnetite,” Appl. Phys. Lett. 98, 182504 (2011). 10.1063/1.3584855

[43] S. de Jong, R. Kukreja, C. Trabant, N. Pontius, C. F. Chang, T. Kachel, M. Beye, F. Sorgenfrei, C. H. Back, B. Bräuer, W. F. Schlotter, J. J. Turner, O. Krupin, M. Doehler, D. Zhu, M. A. Hossain, A. O. Scherz, D. Fausti, F. Novelli, M. Esposito, W. S. Lee, Y. D. Chuang, D. H. Lu, R. G. Moore, M. Yi, M. Trigo, P. Kirchmann, L. Pathey, M. S. Golden, M. Buchholz, P. Metcalf, F. Parmigiani, W. Wurth, A. Föhlisch, C. Schüßler-Langeheine, and H. A. Dürr, “Speed limit of the insulator–metal transition in magnetite,” Nat. Mater. 12, 882–886 (2013). 10.1038/nmat3718 23892787

[44] F. Randi, I. Vergara, F. Novelli, M. Esposito, M. Dell'Angela, V. A. M. Brabers, P. Metcalf, R. Kukreja, H. A. Dürr, D. Fausti, M. Grüninger, and F. Parmigiani, “Phase separation in the nonequilibrium Verwey transition in magnetite,” Phys. Rev. B 93, 054305 (2016). 10.1103/PhysRevB.93.054305

[45] E. Baldini, C. A. Belvin, M. Rodriguez-Vega, I. O. Ozel, D. Legut, A. Kozłowski, A. M. Oleś, K. Parlinski, P. Piekarz, J. Lorenzana, G. A. Fiete, and N. Gedik, “Discovery of the soft electronic modes of the trimeron order in magnetite,” Nat. Phys. 16, 541–545 (2020). 10.1038/s41567-020-0823-y

[46] W. Wang, J. Li, Z. Liang, L. Wu, P. M. Lozano, A. C. Komarek, X. Shen, A. H. Reid, X. Wang, Q. Li, W. Yin, K. Sun, I. K. Robinson, Y. Zhu, M. P. M. Dean, and J. Tao, “Verwey transition as evolution from electronic nematicity to trimerons via electron-phonon coupling,” Sci. Adv. 9, eadf8220 (2023). 10.1126/sciadv.adf8220 37294769 PMC10256157

[47] B. Truc, P. Usai, F. Pennacchio, G. Berruto, R. Claude, I. Madan, V. Sala, T. LaGrange, G. M. Vanacore, S. Benhabib, and F. Carbone, “Ultrafast generation of hidden phases via energy-tuned electronic photoexcitation in magnetite,” Proc. Natl. Acad. Sci. 121, e2316438121 (2024). 10.1073/pnas.2316438121 38900799 PMC11214049

[48] X. Liu, C.-F. Chang, A. D. Rata, A. C. Komarek, and L. H. Tjeng, “Fe_3_O_4_ thin films: Controlling and manipulating an elusive quantum material,” npj Quantum Mater. 1, 16027 (2016). 10.1038/npjquantmats.2016.27

[49] K. Tiedtke, A. Azima, N. von Bargen, L. Bittner, S. Bonfigt, S. Düsterer, B. Faatz, U. Frühling, M. Gensch, C. Gerth, N. Guerassimova, U. Hahn, T. Hans, M. Hesse, K. Honkavaar, U. Jastrow, P. Juranic, S. Kapitzki, B. Keitel, T. Kracht, M. Kuhlmann, W. B. Li, M. Martins, T. Núñez, E. Plönjes, H. Redlin, E. L. Saldin, E. A. Schneidmiller, J. R. Schneider, S. Schreiber, N. Stojanovic, F. Tavella, S. Toleikis, R. Treusch, H. Weigelt, M. Wellhöfer, H. Wabnitz, M. V. Yurkov, and J. Feldhaus, “The soft x-ray free-electron laser FLASH at DESY: Beamlines, diagnostics and end-stations,” New J. Phys. 11, 023029 (2009). 10.1088/1367-2630/11/2/023029

[50] J. Feldhaus, “FLASH—The first soft x-ray free electron laser (FEL) user facility,” J. Phys. B 43, 194002 (2010). 10.1088/0953-4075/43/19/194002

[51] S. Toleikis, “The FLASH facility current status in 2018 and future upgrade plans,” AIP Conf. Proc. 2054, 030015 (2019). 10.1063/1.5084578

[52] M. Beye, R. Y. Engel, J. O. Schunck, S. Dziarzhytski, G. Brenner, and P. S. Miedema, “Non-linear soft x-ray methods on solids with MUSIX—the multi-dimensional spectroscopy and inelastic x-ray scattering endstation,” J. Phys.: Condens. Matter 31, 014003 (2019). 10.1088/1361-648X/aaedf3 30504529

[53] E. L. Saldin, E. A. Schneidmiller, and M. V. Yurkov, “Properties of the third harmonic of the radiation from self-amplified spontaneous emission free electron laser,” Phys. Rev. Spec. Top. - Accel. Beams 9, 030702 (2006). 10.1103/PhysRevSTAB.9.030702

[54] S. Düsterer, P. Radcliffe, G. Geloni, U. Jastrow, M. Kuhlmann, E. Plönjes, K. Tiedtke, R. Treusch, J. Feldhaus, P. Nicolosi, L. Poletto, P. Yeates, H. Luna, J. T. Costello, P. Orr, D. Cubaynes, and M. Meyer, “Spectroscopic characterization of vacuum ultraviolet free electron laser pulses,” Opt. Lett. 31, 1750 (2006). 10.1364/OL.31.001750 16688283

[55] W. Ackermann, G. Asova, V. Ayvazyan, A. Azima, N. Baboi, J. Bähr, V. Balandin, B. Beutner, A. Brandt, A. Bolzmann, R. Brinkmann, O. I. Brovko, M. Castellano, P. Castro, L. Catani, E. Chiadroni, S. Choroba, A. Cianchi, J. T. Costello, D. Cubaynes, J. Dardis, W. Decking, H. Delsim-Hashemi, A. Delserieys, G. Di Pirro, M. Dohlus, S. Düsterer, A. Eckhardt, H. T. Edwards, B. Faatz, J. Feldhaus, K. Flöttmann, J. Frisch, L. Fröhlich, T. Garvey, U. Gensch, C. Gerth, M. Görler, N. Golubeva, H.-J. Grabosch, M. Grecki, O. Grimm, K. Hacker, U. Hahn, J. H. Han, K. Honkavaara, T. Hott, M. Hüning, Y. Ivanisenko, E. Jaeschke, W. Jalmuzna, T. Jezynski, R. Kammering, V. Katalev, K. Kavanagh, E. T. Kennedy, S. Khodyachykh, K. Klose, V. Kocharyan, M. Körfer, M. Kollewe, W. Koprek, S. Korepanov, D. Kostin, M. Krassilnikov, G. Kube, M. Kuhlmann, C. L. S. Lewis, L. Lilje, T. Limberg, D. Lipka, F. Löhl, H. Luna, M. Luong, M. Martins, M. Meyer, P. Michelato, V. Miltchev, W. D. Möller, L. Monaco, W. F. O. Müller, O. Napieralski, O. Napoly, P. Nicolosi, D. Nölle, T. Nuñez, A. Oppelt, C. Pagani, R. Paparella, N. Pchalek, J. Pedregosa-Gutierrez, B. Petersen, B. Petrosyan, G. Petrosyan, L. Petrosyan, J. Pflüger, E. Plönjes, L. Poletto, K. Pozniak, E. Prat, D. Proch, P. Pucyk, P. Radcliffe, H. Redlin, K. Rehlich, M. Richter, M. Roehrs, J. Roensch, R. Romaniuk, M. Ross, J. Rossbach, V. Rybnikov, M. Sachwitz, E. L. Saldin, W. Sandner, H. Schlarb, B. Schmidt, M. Schmitz, P. Schmüser, J. R. Schneider, E. A. Schneidmiller, S. Schnepp, S. Schreiber, M. Seidel, D. Sertore, A. V. Shabunov, C. Simon, S. Simrock, E. Sombrowski, A. A. Sorokin, P. Spanknebel, R. Spesyvtsev, L. Staykov, B. Steffen, F. Stephan, F. Stulle, H. Thom, K. Tiedtke, M. Tischer, S. Toleikis, R. Treusch, D. Trines, I. Tsakov, E. Vogel, T. Weiland, H. Weise, M. Wellhöfer, M. Wendt, I. Will, A. Winter, K. Wittenburg, W. Wurth, P. Yeates, M. V. Yurkov, I. Zagorodnov, and K. Zapfe, “Operation of a free-electron laser from the extreme ultraviolet to the water window,” Nat. Photonics 1, 336–342 (2007). 10.1038/nphoton.2007.76

[56] H. Redlin, A. Al-Shemmary, A. Azima, N. Stojanovic, F. Tavella, I. Will, and S. Düsterer, “The FLASH pump–probe laser system: Setup, characterization and optical beamlines,” Nucl. Instrum. Methods Phys. Res. Sect. A 635, S88–S93 (2011). 10.1016/j.nima.2010.09.159

[57] N. Pontius, M. Beye, C. Trabant, R. Mitzner, F. Sorgenfrei, T. Kachel, M. Wöstmann, S. Roling, H. Zacharias, R. Ivanov, R. Treusch, M. Buchholz, P. Metcalf, C. Schüßler-Langeheine, and A. Föhlisch, “Probing the non-equilibrium transient state in magnetite by a jitter-free two-color X-ray pump and X-ray probe experiment,” Struct. Dyn. 5, 054501 (2018). 10.1063/1.5042847 30310825 PMC6158032

[58] C. F. Chang, J. Schlappa, M. Buchholz, A. Tanaka, E. Schierle, D. Schmitz, H. Ott, R. Sutarto, T. Willers, P. Metcalf, L. H. Tjeng, and C. Schüßler-Langeheine, “Intrinsic and extrinsic x-ray absorption effects in soft x-ray diffraction from the superstructure in magnetite,” Phys. Rev. B 83, 073105 (2011). 10.1103/PhysRevB.83.073105

[59] T. Tschentscher, C. Bressler, J. Grünert, A. Madsen, A. Mancuso, M. Meyer, A. Scherz, H. Sinn, and U. Zastrau, “Photon Beam Transport and Scientific Instruments at the European XFEL,” Appl. Sci. 7, 592 (2017). 10.3390/app7060592

[60] R. Y. Engel, O. Alexander, K. Atak, U. Bovensiepen, J. Buck, R. Carley, M. Cascella, V. Chardonnet, G. S. Chiuzbaian, C. David, F. Döring, A. Eschenlohr, N. Gerasimova, F. de Groot, L. L. Guyader, O. S. Humphries, M. Izquierdo, E. Jal, A. Kubec, T. Laarmann, C.-H. Lambert, J. Lüning, J. P. Marangos, L. Mercadier, G. Mercurio, P. S. Miedema, K. Ollefs, B. Pfau, B. Rösner, K. Rossnagel, N. Rothenbach, A. Scherz, J. Schlappa, M. Scholz, J. O. Schunck, K. Setoodehnia, C. Stamm, S. Techert, S. M. Vinko, H. Wende, A. A. Yaroslavtsev, Z. Yin, and M. Beye, “Electron population dynamics in resonant non-linear x-ray absorption in nickel at a free-electron laser,” Struct. Dyn. 10, 054501 (2023). 10.1063/4.0000206 37841290 PMC10576398

[61] See the 10.60893/figshare.sdy.c.7705769 for some more detailed information on the calibration of the pump laser fluences, experimental parameters (temporal resolution of the setup, Fresnel zone plate optic, and detector), and the fitting procedure of the pump-probe delay traces.

